# A Case of Erythema Nodosum with Coccidioidomycosis

**DOI:** 10.5811/cpcem.2017.3.33177

**Published:** 2017-07-14

**Authors:** Saema Said, Justin Yanuck, Michael J. Burns

**Affiliations:** University of California Irvine School of Medicine, Department of Emergency Medicine, Irvine, California

## Abstract

Erythema nodosum (EN) is associated with many systemic diseases and infections. This case report provides an image of erythematous nodules, an overview of the various causes of EN, and the laboratory tests and imaging that can be done in the emergency department to narrow its broad differential diagnosis.

## CASE PRESENTATION

A 26-year-old male presented to an emergency department (ED) in Southern California with two days of symmetrical, erythematous, and painful subcutaneous nodules. The nodules began on his thighs and spread to his anterior lower legs (Image). He also complained of two weeks of fever, cough, arthralgia, generalized weakness, and dyspnea, and had right upper-lobe consolidation and mediastinal adenopathy on imaging. He had been seen in the ED two weeks earlier for these symptoms and discharged with azithromycin. Since his initial visit, he had no improvement of symptoms with azithromycin and had newly developed leg lesions. Further evaluation as an inpatient confirmed the diagnosis of erythema nodosum (EN) caused by acute coccidoidomycosis. He was started on fluconazole with outpatient follow-up.

## DISCUSSION

EN is a panniculitis resulting from a delayed hypersensitivity reaction.^1^ It presents as tender, warm, and erythematous nodules, usually on the anterior legs.^2^ The nodules are symmetric and 1–5 cm in diameter.^1^ EN is often preceded 1–3 weeks by a prodrome of fever, malaise, arthralgia, cough, and weight loss.^3^ Additional signs and symptoms vary depending on etiology. Although most cases are idiopathic, EN is also associated with various infections (*Streptococcus pyogenes*, yersiniosis, *Chlamydia*, histoplasmosis, coccidioidomycosis, tuberculosis, *Campylobacter*), drugs (estrogens/oral contraceptives, sulfonamides, penicillin), systemic illnesses such as inflammatory bowel disease and sarcoidosis, pregnancy, and malignancy.^3^ Differential diagnosis includes α_1_-antitripsin deficiency, cytophagic histiocytic panniculitis, lupus panniculitis, and nodular fat necrosis.^4^ Tests performed in the workup of EN should depend on the patient’s geographic location, travel history, and presenting symptoms.^4^ For example, patients with EN and upper respiratory symptoms in the western United States should be tested for coccidioidomycosis. EN is self-limited; however, nonsteroidal anti-inflammatory drugs and measures, such as leg rest, elevation, and compression, can reduce pain and edema.^1^,^3^ Definitive management is treatment of underlying trigger.

CPC-EM Capsule

What do we already know about this clinical entity?

Erythema nodosum (EN) presents as tender, erythematous cutaneous nodules. It can be idiopathic or associated with various infections, systemic diseases, and drugs.

What is the major impact of the image(s)?

The image in this case report demonstrates the classic findings seen with EN: symmetrical, erythematous nodules on anterior lower legs.

How might this improve emergency medicine practice?

Given the broad spectrum of conditions associated with EN, this case report addresses how to recognize and efficiently workup EN in the emergency department.

## Figures and Tables

**Image:**
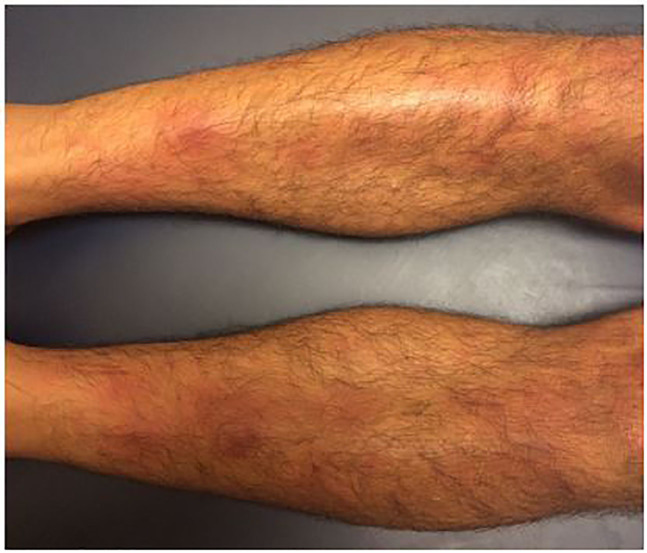
Erythematous nodules on bilateral anterior legs.
